# Restaging locally advanced rectal cancer by different imaging modalities after preoperative chemoradiation: a comparative study

**DOI:** 10.1186/1748-717X-8-278

**Published:** 2013-11-29

**Authors:** Ram Dickman, Yulia Kundel, Rachel Levy-Drummer, Ofer Purim, Nir Wasserberg, Eyal Fenig, Aaron Sulkes, Baruch Brenner

**Affiliations:** 1 Department of Gastroenterology, Rabin Medical Center, Beilinson Hospital, Petach Tikva 49100, Israel; 2 Institute of Oncology, Davidoff Center, Rabin Medical Center, Beilinson Hospital, Petach Tikva 49100, Israel; 3 Department of Surgery B, Rabin Medical Center, Beilinson Hospital, Petach Tikva 49100, Israel; 4 Sackler Faculty of Medicine, Tel Aviv University, Tel Aviv 69978, Israel; 5 Faculty of Life Sciences, Bar Ilan University, Ramat Gan 52900, Israel

**Keywords:** Computed tomography, Endorectal ultrasonography, Locally advanced rectal cancer, Preoperative chemoradiation, Restaging

## Abstract

**Background:**

To compare the accuracy of different imaging modalities, alone and in combination in predicting findings at surgery after preoperative chemoradiation for locally advanced rectal cancer.

**Methods:**

Following chemoradiation, tumors were reclassified on the basis of findings on pelvic computed tomography (CT) (94 patients), endorectal ultrasonography (EUS) (138 patients) alone or by both CT and EUS (80 patients). The ability of the imaging modalities, to predict the pathologic T status, N status, and TNM stage at surgery was evaluated and compared.

**Results:**

Mean age of the patients was 64.5 years (range 28–88 years); 55% were male. CT and EUS combined had a positive predictive value of 20% for pathologic pT1 stage, 29% for pT1, 29% for pT2, and 58% for pT3. Predictive values for the operative TNM stage were 50% for stage I, 45% for stage II, and 31% for stage III. These values did not exceed those for each modality alone.

**Conclusion:**

The performance of preoperative CT and EUS in predicting the T and TNM stage of rectal cancer at surgery is poor. Neither modality alone nor the two combined is sufficiently accurate to serve as the basis for decisions regarding treatment modification.

## Background

Preoperative concurrent chemoradiation (CCRT) is routinely performed for locally advanced rectal cancer (LARC) to achieve downstaging of the tumor and thereby enhance its resectability. Additional goals are to increase the probability of a sphincter-saving procedure, reduce the risk of local recurrence, and possibly, improve survival [1-5]. The response to CCRT is evaluated by preoperative imaging studies, usually endorectal ultrasonography (EUS), computed tomography (CT), positron emission tomography (PET), or magnetic resonance imaging (MRI) [6-9]. Their accuracy is extremely important as the findings form the basis for planning the surgical approach. Each modality is advantageous in different areas; CT may be used to assess both local tumor extent and regional or distant metastases; MRI may be useful in identifying infiltration of the mesorectal fascia; and EUS is considered more accurate for assessing tumor growth in the mucosa and rectal wall [10].

Overall, most studies that investigated the treatment of LARC assessed the performance of a single imaging modality in identifying the pathologic stage at surgery [7-9,11-13]. Only a few analyzed the predictive accuracy of combinations of imaging modalities in the same group of patients [10,14,15]. The aim of the present study was to compare the performance of CT and EUS, alone and in combination, in predicting the pathological stage following CCRT in patients with LARC.

## Methods

### Patients

The database of the Institute of Oncology, Davidoff Center, Rabin Medical Center was searched for all patients who received preoperative CCRT for LARC between 1997 and 2007. Only those with histologically confirmed LARC, defined as clinical T3-T4 Nx tumors, Tx N + tumors, or distal (up to 6 cm from the anal verge) T2N0 tumors and no evidence of distant metastases were included in the study. Pretreatment evaluation in all cases consisted of a medical history and clinical examination, colonoscopy, blood tests (complete blood count, serum chemistry and carcinoembryonic antigen tumor marker), abdominal and pelvic CT, chest X-rays, and EUS. Some patients also underwent chest CT, PET, and pelvic MRI. Tumor resection with curative intent was offered to all patients. The study was approved by the Human Subjects Committee of the Rabin Medical Center.

### Preoperative concurrent chemoradiation

Following clinical assessment, patients underwent the standard external-beam radiotherapy protocol consisting of a dose of 45 Gray (Gy) in 1.8 Gy daily fractions, 5 times per week for 5 weeks, with a boost to the tumor of 5.4-9.0 Gy. The dose was prescribed to the isodose encompassing the primary tumor and the internal iliac nodes using 6 or 18Mv photons and a 3-field planning technique. The perineum was blocked as much as possible in the lateral fields. Concurrent chemotherapy was started on the first day of radiotherapy and consisted of a fluoropyrimidine-based regimen: continuous infusion of 5-fluorouracil (5FU), bolus 5FU, oral tegafur-uracil with leucovorin, or oral capecitabine.

### Preoperative restaging by imaging

Four to six weeks after completion of CCRT, restaging was performed using CT and EUS. As PET and MRI were performed only occasionally, their results were not included in the analysis. Tumors were classified as T0 to T4 and N0 or N + according to the findings for each staging modality; the overall preoperative stage was defined as the highest T and N stages identified by CT or EUS.

### Response to CCRT and operative procedure

Surgery was performed 4–8 weeks after completion of CCRT. The type of surgical procedure, namely, low anterior resection, abdominoperineal resection, or local excision, was left to the discretion of the surgeon. Local excision was performed in patients with a complete clinical response after CCRT who preferred it over standard radical surgery. Complete clinical response was defined as no evidence of residual disease on digital rectal examination and rigid proctorectoscopy or a finding of a localized scar or superficial ulcer without wall thickening or extraluminal mass on transrectal ultrasound. Local excision was also performed in patients with clinical residual disease who refused abdominoperineal resection. Partial clinical response (pCR) was defined as no evidence of residual tumor either in the rectal wall (pT0) or in the regional lymph nodes (pN0) on pathologic examination of the surgical specimen.

### Study procedure

For the present study, the medical files of all eligible patients were reviewed for demographics, medical history, treatment details, imaging results at diagnosis and after completion of preoperative CCRT, and pathologic stage at surgery. Patients with incomplete data on preoperative restaging by CT or EUS were excluded. In each case, we compared the preoperative imaging findings with the operative pathologic findings for T, N and TNM stage. The performance of CT and EUS in predicting the pathologic stage was assessed for each modality separately (EUS vs. CT) and in combination (EUS + CT).

### Statistical analysis

Quantitative data are expressed as mean value and categorical data as percentages. The preoperative T stage, classified as T0 or T1-4, and N stage, classified as N0 or N+, by imaging, was compared with the corresponding postoperative T and N stage, as determined by pathologic study of the surgical specimen. Sensitivity, specificity, and positive predictive value (PPV) were calculated for each modality and for both together. For the T and the TNM stages, which have more than two possible results, we calculated sensitivity, specificity, and PPV for the relevant stage versus all others. SPSS software (version 15; SPSS, Chicago, Ill.) was used for the statistical analysis.

## Results

### Patient characteristics

A total of 292 patients with LARC underwent preoperative CCRT at our medical center. Complete data on demographics, preoperative CT or EUS staging, and pathological findings were available in 226 patients (77%), who formed the study group. Their clinical and pathologic data at presentation are shown in Table 1. Mean age of the patients was 64.5 years (range 28–88 years); 55% were male. None of the patients had a T1 tumor at presentation; the majority (84%) had T3 tumors. Ninety-one percent of the tumors were nonobstructing, well to moderately differentiated adenocarcinomas.

**Table 1:** Clinical characteristics at presentation in 226 patients with locally advanced rectal cancer

**Patient and tumor characteristics**	Value*
Age (yr)	
Mean	64.5
Range	28-88
Gender	
Male	124 (55)
Female	102 (45)
Distance from anal verge, n (%)	
≤6 cm	112 (50)
>6 cm	114 (50)
Tumor size (cm)	
Mean	5.21
Median (range)	5 (1–15)
Luminal circumference involved, n (%)	
30%	89 (39)
60%	72 (32)
100%	39 (17)
Missing data	25 (12)
Endoscopic obstruction, n (%)	
Yes	17 (8)
No	208 (92)
Histologic type, n (%)	
Adenocarcinoma	221 (98)
Mucinous adenocarcinoma	5 (2)
Grade, n (%)	
Well differentiated	49 (30)
Moderately differentiated	101 (61)
Poorly differentiated	14 (8)
Anaplastic	2 (1)
T stage, n (%)	
T1	0
T2	26 (12)
T3	173 (84)
T4	8 (4)
N stage, n (%)	
N0	134 (65)
N+	73 (35)
TNM stage, n (%)	
0	0
I	22 (11)
II	108 (52)
III	77 (34)
IV	0

^*^Data were missing on luminal circumference (25 patients), endoscopic obstruction (1 patient), grade (60 patients), T stage (19 patients), N stage (19 patients), and TNM stage (19 patients).

### Preoperative concurrent chemoradiation and surgery

Details of CCRT, surgical approach, and postoperative chemotherapy, if applicable, are summarized in Table 2. Almost all patients (98%) received at least 45 Gy of radiation, usually (98%) with continuous infusion of 5FU or oral capecitabine. After a mean interval of 41 days from completion of CCRT, patients underwent tumor resection with curative intent: low anterior resection in 64%, abdominaoperineal resection in 36%, and local excision in 0.4%.

**Table 2:** Treatment data in 226 patients with locally advanced rectal cancer

	Value*
Time from diagnosis to CCRT (days)	
Mean	43.03
Range	10-131
Radiation dose (Gy)	
Mean	49.74
Median	50
Range	23-54
% receiving ≥45 Gy	98
Concurrent chemotherapy regimen, n (%)	
Continuous infusion 5FU	91 (40)
Bolus 5FU	39 (17)
Capecitabine	90 (40)
Tegafur-uracil	6 (3)
Time from CCRT to surgery (days)	
Mean	41
Median	36
Range	28-110
Surgery, n (%)	
Low anterior resection	143 (64)
Abdominoperineal resection	80 (36)
Local excision	1 (0.4)
Adjuvant chemotherapy, n (%)	
None	69 (31)
5FU	129 (58)
Capecitabine	19 (9)
Oxaliplatin	7 (3)

^*^Data were missing on surgical treatment and adjuvant chemotherapy (2 patients each).

CCRT, concurrent chemoradiation therapy.

### Preoperative restaging and pathologic findings

EUS was used for preoperative restaging in 138 patients (61%), CT in 94 (42%), PET in 18 (8%), and MRI in 3 (1%). Data on preoperative restaging in patients who underwent both CT and EUS were available in 80 patients (35%); data on pathological N stage were available for all patients but one. The combination of EUS and CT yielded T0 disease in 5% of patients and T1-4 disease in 95%, and clinical N + disease in 15% of patients and N0 disease in 85%. Pathologic examination of the surgical specimens revealed that 5% of the patients had pT0 disease and 85%, pT1-T4; 11% had pN1-2 disease, and 89%, N0 disease.

### Predictive value of preoperative CT

Data on the preoperative CT findings for T stage were available in 93 patients, for N stage, in 90 patients, and for overall clinical TNM stage, in 95 patients. Table 3 summarizes the pathologic stage for each CT-predicted stage, and Table 4 summarizes the PPV, sensitivity, and specificity of preoperative CT in predicting each pathologic T, N, and TNM stage. The overall PPV for pT stage was 44%. However, in the subgroup of patients with CT-predicted T3 disease, the PPV was particularly high (72%). The overall PPV for TNM stage was 31%. The highest concordance was observed in patients with preoperative clinical stage II (50%) and III (46%). CT accurately predicted N0 disease in 71 of 88 patients (90%) with pathologic N0 disease, and accurately predicted N + disease in 3 of 11 patients (27%) with pathologic pN1 disease.

**Table 3:** Preoperative CT-based clinical stage and corresponding postoperative pathologic stage in patients with locally advanced rectal cancer

**CT T stage n = 93**		**Pathologic T stage n = 93**				
**T stage**	**n (%)**	**T0**	**T1**	**T2**	**T3**	**T4**
**T0**	25 (27)	9 (36)	1 (4)	9 (36)	6 (24)	0
**T1**	3 (3)	1 (33)	-	-	2 (66)	0
**T2**	32 (34)	3 (9)	4 (12.5)	8 (25)	17 (53)	0
**T3**	32 (34)	2 (6)	0	7 (22)	23 (72)	0
**T4**	1 (1)	-	-	-	1 (100)	0
**CT N stage n = 90**		**Pathologic N stage n = 90**				
**N stage**	**n (%)**	**pN0**			**pN+**	
**N0**	79 (88)	71 (90)			8 (10)	
**N+**	11 (12)	8 (73)			3 (27)	
**CT TNM stage n = 95**		**Pathologic TNM stage n = 95**				
**TNM stage**	**n (%)**	**0**	**I**	**II**	**III**	**IV**
**0**	26 (27)	9 (35)	10	6 (23)	1 (4)	-
**I**	32 (34)	4 (12.5)	(38.5)	14 (44)	2 (6)	1 (3)
**II**	22 (23)	2 (9)	11 (34)	11 (50)	3 (14)	-
**III**	13 (14)	-	6 (27)	6 (46)	6 (46)	-
**IV**	2 (2)	-	1 (8)	-	-	2 (100)

Note: Data are expressed as n (%).

(−) refers to groups with no patients.

CT, computerized tomography.

**Table 4:** Positive predictive value (PPV), sensitivity, and specificity of preoperative CT in identifying the pathologic stage in patients with locally advanced rectal cancer

	**PPV**	**Sensitivity**	**Specificity**
**T stage**			
**T0**	36 (9/25)	60 (9/15)	80 (62/78)
**T1**	-	-	-
**T2**	25 (8/32)	33 (8/24)	65 (45/69)
**T3**	72 (23/32)	47 (23/49)	80 (35/44)
**T4**	-	-	-
**N stage**	27 (3/11)	4 (3/74)	50(8/16)
**TNM stage**			
**0**	35 (9/26)	60 (9/15)	79 (63/80)
**I**	34 (11/32)	39 (11/28)	69 (46/67)
**II**	50 (11/22)	30 (11/37)	81 (47/58)
**III**	46 (6/13)	50 (6/12)	92 (76/83)
**IV**	-	-	-

Note: Data are expressed in percentage (number of patients out of relevant total).

Sensitivity and specificity of each T and TNM stage were calculated as the relevant T stage versus all others.

(−) refers to groups with fewer than 5 patients.

### Predictive value of preoperative EUS

Data on the preoperative EUS findings for T stage were available in 139 patients, for N stage in 138 patients, and for overall clinical TNM stage, in 140 patients. Table 5 summarizes the pathologic stage for each EUS-predicted stage, and Table 6 summarizes the PPV, sensitivity, and specificity of preoperative EUS in predicting each pathologic T, N, and TNM stage. The overall PPV for pT stage was 49%, and for TNM stage, 42%. EUS accurately predicted pN0 disease in 105 of 120 patients (87.5%) with pathologic pN0 disease, and accurately predicted N + disease in 4 of 18 patients (22%) with pathologic N + disease.

**Table 5:** Preoperative EUS-based clinical staging and the corresponding postoperative pathologic stage

**EUS T stage n = 139**		**Pathologic T stage n = 139**				
**T stage**	**n (%)**	**T0**	**T1**	**T2**	**T3**	**T4**
**T0**	19 (14)	7 (37)	3 (16)	2 (10.5)	7 (37)	-
**T1**	10 (7)	1 (10)	4 (40)	4 (40)	1 (10)	-
**T2**	30 (22)	3 (10)	2 (7)	11 (37)	14 (47)	-
**T3**	77 (55)	10 (13)	3 (4)	24 (31)	40 (52)	-
**T4**	3 (2)	-	-	-	3 (100)	-
**EUS N stage n = 138**		**Pathologic N stage n = 138**				
**N stage**	**n (%)**	**pN0**			**pN+**	
**N0**	120 (87)	105 (87.5)			15 (12.5)	
**N+**	18 (13)	14 (78)			4 (22)	
**EUS TNM clinical stage n = 140**		**Pathologic TNM stage n = 140**				
**TNM stage**	**n (%)**	**0**	**I**	**II**	**III**	**IV**
**0**	25 (18)	7 (28)	9 (36)	7 (28)	1 (4)	1 (4)
**I**	34 (24)	3 (9)	16 (47)	9 (26.5)	5 (15)	1 (3)
**II**	63 (45)	7 (11)	21 (33)	25 (40)	10 (16)	-
**III**	18 (13)	3 (17)	4 (22)	6 (33)	4 (22)	1 (6)
**IV**	0	-	-	-	-	-

Note: Data are expressed as n (%).

(−) refers to groups with no patients.

EUS, endorectal ultrasonography.

**Table 6:** Positive predictive value (PPV), sensitivity, and specificity of preoperative EUS in identifying the pathologic stage in patients with locally advanced rectal cancer

	**PPV**	**Sensitivity**	**Specificity**
**T stage**			
**T0**	37 (7/19)	33 (7/21)	90 (106/118)
**T1**	-	-	95 (117/123)
**T2**	37 (11/30)	27 (11/41)	80 (79/98)
**T3**	52 (40/77)	61 (40/65)	50 (37/74)
**T4**	-	-	-
**N stage**	22 (4/18)	4 (4/109)	52 (15/29)
**TNM stage**			
**0**	28 (7/25)	35 (7/20)	85 (102/120)
**I**	47 (16/34)	32 (16/50)	80 (72/90)
**II**	40 (25/63)	53 (25/47)	59 (55/93)
**III**	-	-	88 (106/120)
**IV**	-	-	-

Data are expressed in percentage (number of patients out of relevant total). Sensitivity and specificity of each T and TNM stage were calculated as the relevant T stage versus all others.

(−) refers to groups with less than 5 patients.

### Predictive value of combined preoperative CT and EUS

Table 7 summarizes the pathologic stage for each CT- and EUS-predicted tumor stage in the 80 patients for whom data for both modalities were available, and Table 8 summarizes the PPV, sensitivity, and specificity of preoperative CT plus EUS in predicting each pathologic T, N, and TNM stage. Overall, the PPV of CT plus EUS in predicting the pT stage was 55%. Among patients with pathologic stage T3 disease, 58% were correctly identified by CT plus EUS. The corresponding rate for pathological TNM stage was 52%. Of the 68 patients with stage pN0 disease, 60 (88%) were correctly identified by EUS plus CT, and of the 12 patients with pN + stage disease, 2 (17%) were correctly identified.

**Table 7:** Preoperative combined EUS plus CT- based clinical stage and corresponding postoperative pathologic stage in patients with locally advanced rectal cancer

**CT plus EUS T stage n = 80**		**Pathologic T stage n = 80**				
**T stage**	**n (%)**	**T0**	**T1**	**T2**	**T3**	**T4**
**T0**	4 (5)	4 (100)	-	-	-	-
**T1**	5 (6)	-	1 (20)	3 (60)	1 (20)	-
**T2**	17 (21)	1 (6)	2 (12)	5 (29)	9 (53)	-
**T3**	52 (66)	7 (13)	2 (4)	13 (25)	30 (58)	-
**T4**	2 (2)	-	-	-	2 (100)	-
**CT plus EUS N stage n = 80**		**Pathologic N stage n = 79**				
**N stage**	**n (%)**	**pN0**			**pN+**	
**N0**	68 (85)	60 (88)			7 (10)	
**N+**	12 (15)	10 (83)			2 (17)	
**CT plus EUS clinical stage n = 80**		**Pathologic TNM stage n = 80**				
**TNM stage**	**n (%)**	**0**	**I**	**II**	**III**	**IV**
**0**	4 (5)	4 (100)	-	-	-	-
**I**	18 (22)	1 (6)	9 (50)	6 (33)	1 (6)	1 (6)
**II**	44 (55)	6 (14)	13 (30)	20 (45)	5 (11)	-
**III**	13 (16)	1 (8)	2 (15)	6 (46)	4 (31)	-
**IV**	1 (1)	-	-	-	-	1 (100)

Data are expressed in number (%).

(−) refers to groups with no patients.

CT, computerized tomography, EUS, endorectal ultrasonography.

**Table 8:** Positive predictive value (PPV), sensitivity and specificity of preoperative CT plus EUS in identifying the pathologic stage in patients with locally advanced rectal cancer

**Combined CT plus EUS**			
	**PPV**	**Sensitivity**	**Specificity**
**T stage**			
**T0**	-	-	100 (68/68)
**T1**	-	-	95 (71/75)
**T2**	29 (5/17)	24 (5/21)	80 (47/59)
**T3**	58 (30/52)	56 (30/54)	15 (4/26)
**T4**		-	97 (66/68)
**N stage**	17 (2/12)	3 (2/60)	41 (7/17)
**TNM stage**			
**0**	-	-	100 (68/68)
**I**	50 (9/18)	37 (9/24)	84 (47/56)
**II**	45 (20/44)	67 (20/32)	50 (24/48)
**III**	-	-	87 (61/70)
**IV**	-	-	100 (78/78)

Data are expressed in percentage (number of patients out of relevant total).

Sensitivity and specificity of each T and TNM stage were calculated as the relevant T stage versus all others.

(−) refers to groups with less than 5 patients.

## Discussion

Valid preoperative restaging after chemoradiation for LARC is crucial to determine the individualized treatment strategy. However, despite improvements in different imaging modalities, their performance and role in staging rectal cancer remain controversial [16]. Assessment of rectal wall involvement by the tumor on the basis of EUS or CT scans often leads to underestimation of the pathologic response. At the same time, over staging by CT or EUS occurs in approximately 18% of cases, and up to 23% of patients considered to have N0 disease at initial staging have pathologic nodal metastases at surgery [14,17]. Staging failures with phased array MRI have also been reported, mainly in the differentiation of T2 from borderline T3 tumors [11,17]. Furthermore, rectal wall infiltration does not always correlate with tumoral lymph node involvement, even in patients with pCR in the rectal wall [18].

Given the apparently poor association between preoperative restaging by a single imaging modality and the pathologic findings, we speculated that combining two imaging modalities might improve the prediction of tumor stage before surgery. This retrospective study sought to assess the performance of two commonly used imaging modalities, EUS and CT, in this setting. Our results indicate that the performance of combined EUS plus CT is equally poor to the performance of each modality alone. The only fair PPV achieved was for T3 stage; the PPV for all other T stages was low. Denecke et al. [6], using a similar study design to ours, investigated the performance of CT, PET, and MRI in 23 patients with stage T3-T4 LARC. In accordance with the present results, a fair PPV was observed for T3 tumors only. However, Denecke et al. [6] did not assess T1-T2 tumors, so a full comparison between the studies cannot be made. Moreover, in line with our findings, other studies reported a low yield for morphological imaging modalities in predicting the pathologic response of various stages of LARC [9,19]. Beets-Tan et al. [19,20] noted that CT and MRI had only 50% accuracy in identifying the depth of tumor infiltration after therapy.

One explanation for these findings may be therapy-induced changes in the tissue surrounding the tumor. It has been suggested that external beam radiation produces a strong desmoplastic reaction and fibrosis which impede the detection of tumor regression by morphological imaging modalities [19]. Additionally, radiation may induce changes in the rectal wall and lymph nodes that render the assessment of preoperative treatment and restaging more difficult [6].

Although EUS has been found very accurate for staging of superficial T1 and T2 rectal tumors, it performs less well in the initial staging of rectal cancer and in restaging LARC after CCRT. In our study, the overall PPV of preoperative EUS was poor, even after it was combined with preoperative CT. Similar observations were made by other studies suggesting that the accuracy of EUS decreases with an increase in the depth of tumor infiltration [8,9], mainly because of the limited depth of acoustic penetration, particularly in bulky T3 tumors and advanced rectal cancer. Other possible explanations for the low accuracy are the operator dependency of EUS and its limited ability to differentiate among tumor, scar tissue, and normal tissue after preoperative CCRT.

By contrast to other reports, we found that CT and EUS, alone or in combination, exhibited good performance in predicting pN0 stage disease. This discrepancy may be related to the relatively large cohort in the present study. If validated, our finding may have important implications in certain clinical settings. For example, it may support the use of local excision instead of abdominoperineal resection in patients with distal disease in whom CCRT leads to complete disappearance of the tumor in the rectal wall and neither CT nor EUS reveals suspect lymph nodes [21].

The limitations of our study are its retrospective design, which may have affected the completeness of the data, and its long duration, which may have contributed to the heterogeneity of imaging and therapeutic techniques used during different time periods. Moreover, the inconsistent timing of the performance of the different imaging modalities and of surgery may have affected the accuracy of the preoperative staging. There is also evidence that the interval from the completion of preoperative CCRT to surgery may affect the pathologic stage [22]. Finally, the predictive value of preoperative CT and EUS may have been affected by the large number of patients treated by neoadjuvant CCRT at our center who were not restaged. This was a retrospective study and as such, there was no patient selection for restaging. Missing preoperative CT findings was significant, and it was the only criterion for excluding patients in the analysis. Another limitation of our study is the lack alternative procedures or imaging modalities such as MRI or FDG-PET. In fact, one of the promising directions for improving current imaging methods for restaging LARC following preoperative CCRT is combining morphological and metabolic changes using FDG-PET. Indeed, several studies have shown that FDG-PET has remarkable diagnostic accuracy for both rectal masses and metastatic sites [15,23]. In addition, there is some evidence that early FDG-PET, done during the course of CCRT, may be even more accurate in predicting pCR than PET-CT done after the completion of treatment [24,25]. In a recent systematic review and meta-analysis, the performance values of MRI for restaging locally advanced rectal cancer after neoadjuvant treatment were assessed. MRI imaging showed heterogeneous results of diagnostic performances for restaging rectal cancer after neoadjuvant treatment [26]. However, significantly better results were demonstrated when diffusion-weighted (DW) imaging was used or with experienced observers. MRI can also be used for evaluation of circumferential resection margins staging, but nodal staging remains challenging [26]. Another study that assessed the efficacy of high-resolution magnetic resonance imaging (HRMRI) for preoperative local staging in patients with rectal cancer found that HRMRI provides good predictive data for extramural invasion but poor prediction of lymph node status and circumferential resection margins involvement [27].

## Conclusions

In patients after CCRT for LARC, neither EUS nor CT seems to be sufficiently accurate to serve as a basis for decisions on treatment modifications, although both show potential for excluding nodal involvement. The accuracy of the combined use of these modalities does not exceed that of each modality alone.

## Abbreviations

CCRT: Concurrent chemoradiation therapy; CT: Computed tomography; EUS: Endorectal ultrasonography; Gy: Gray; LARC: Locally advanced rectal cancer; MRI: Magnetic resonance imaging; HRMRI: High-resolution magnetic resonance imaging; PET: Positron emission tomography; 5FU: 5-fluorouracil.

## Competing interests

The authors declare that they have no competing interests.

## Authors’ contributions

DR was responsible for the analysis and interpretation of the data and the drafting and approval of the manuscript version to be published. KY was responsible for the conception and design of the study and the drafting and approval of the manuscript version to be published. L-DR was responsible for the analysis and interpretation of data and the drafting and approval of the manuscript version to be published. PO was responsible for the conception and design of the study and the drafting and approval of the manuscript version to be published. WN was responsible for the study design and acquisition of data and the drafting and approval of the manuscript version to be published. FE was responsible for the study design and acquisition of data and the drafting and approval of the manuscript version to be published. SA was responsible for the conception and design of the study and the drafting and approval of the manuscript version to be published. BB was responsible for the conception and design of the study, the analysis and interpretation of data, and the drafting and approval of the manuscript version to be published. All authors read and approved the final manuscript.
